# A new method of rendezvous technique – catheter assistance system: report of a video case at balloon-endoscopy-assisted endoscopic retrograde cholangiopancreatography

**DOI:** 10.1055/a-2744-8675

**Published:** 2025-12-11

**Authors:** Kazunori Nagashima, Manabu Ishikawa, Tomoya Sakamoto, Koh Fukushi, Tsunehiro Suzuki, Toshimitu Murohisa, Atsushi Irisawa

**Affiliations:** 1 12756 Department of Gastroenterology, Dokkyo Medical University School of Medicine, Mibu, Japan; 2 46624 Japanese Red Cross Ashikaga Hospital, Ashikaga, Japan

Two useful rendezvous methods are used during endoscopic retrograde cholangiopancreatography (ERCP): (1) along-the-wire method and (2) over-the-wire method 1 2 3 . Especially, for balloon-endoscopy-assisted ERCP (BE-ERCP) in patients with altered gastrointestinal anatomy, cannulation might be difficult even with availability of the two rendezvous methods. We developed a method of rendezvous technique, a catheter assistance system (CAS), that combines over-the-wire method with a catheter. To the best of our knowledge, this is the first video case of the CAS used for BE-ERCP with altered gastrointestinal anatomy.

This video presents a typical case ( Video 1 ). A 79-year-old woman with obstructive jaundice associated with bile duct stricture ( Fig. 1 ) had prior gastric cancer surgery. Cannulation for that BE-ERCP was difficult; bile duct drainage was unsuccessful. Therefore, PTBD was conducted. Two weeks later, the CAS was performed through the fistula-forming PTBD route. A guidewire and the ERCP standard catheter (MTW; ABIS Inc., Japan, Tokyo) were inserted and placed in the intestine through a percutaneous route ( Fig. 2 ). The catheter tip was grasped with a snare and pulled into the endoscope ( Fig. 3 ). When the catheter exited the endoscopic channel, the guidewire was pulled out from the percutaneous route side ( Fig. 4 ). A guidewire was inserted from the catheter tip toward the bile duct. The catheter alone was removed from the percutaneous route while retaining the wire. A guidewire was detained in the bile duct, from which the usual ERCP procedure was performed ( Fig. 5 ). Drainage was performed with no adverse events.

This report is the first of a video case for which CAS was performed for BE-ERCP. This treatment can realize safer and more reliable rendezvous methods, especially when performing BE-ERCP. BE-ERCP, balloon-endoscopy-assisted endoscopic retrograde cholangiopancreatography; CAS, catheter assistance system.Video 1

**Fig. 1:**
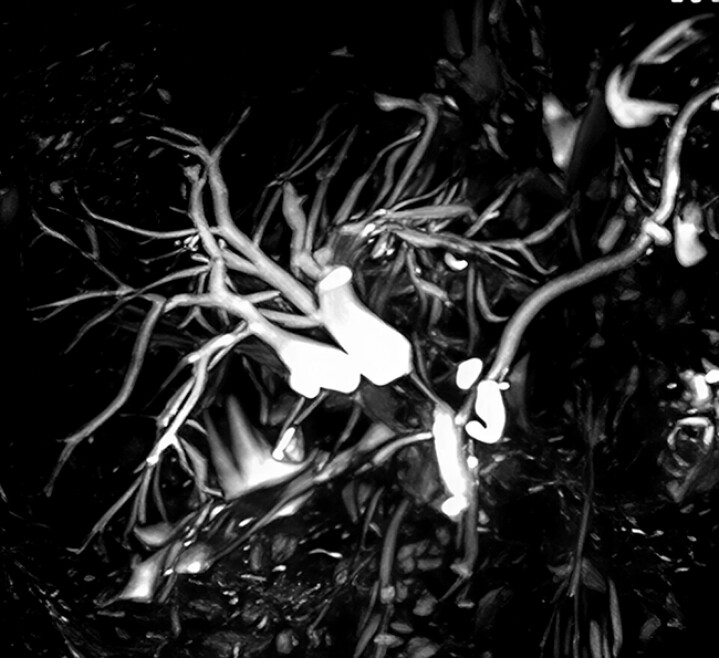
This MRCP image shows bile duct stenosis.

**Fig. 2:**
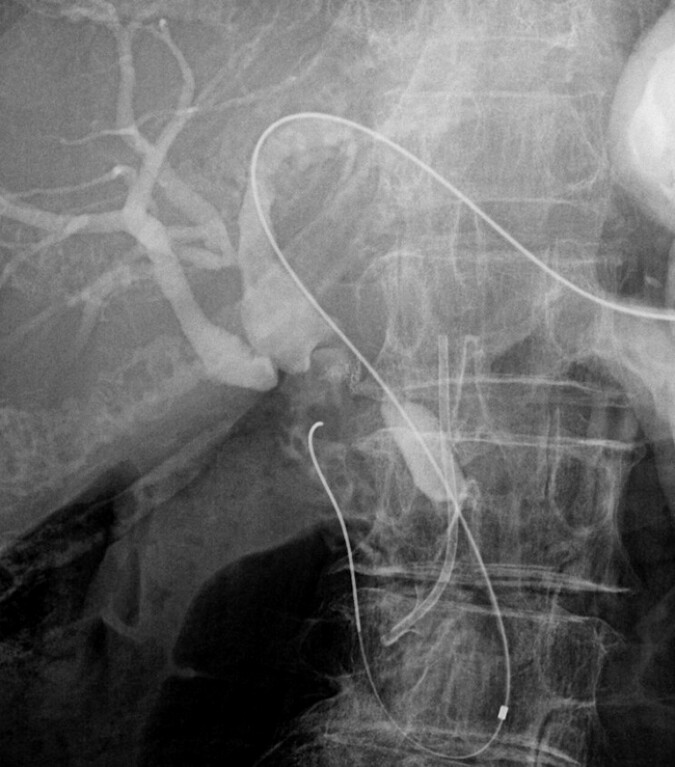
A guidewire and the ERCP standard catheter (MTW; ABIS Inc., Japan, Tokyo) are inserted and placed in the intestine. ERCP, endoscopic retrograde cholangiopancreatography.

**Fig. 3:**
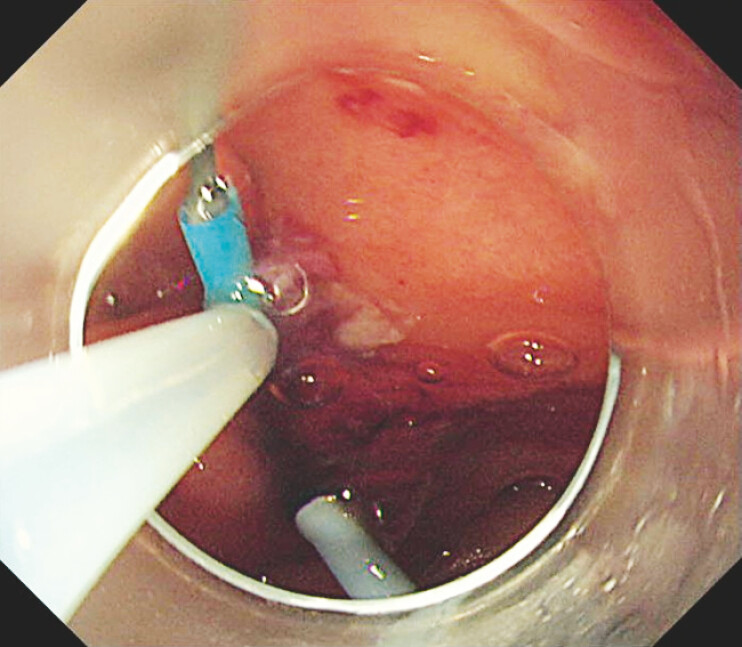
The catheter was pulled into the endoscope.

**Fig. 4:**
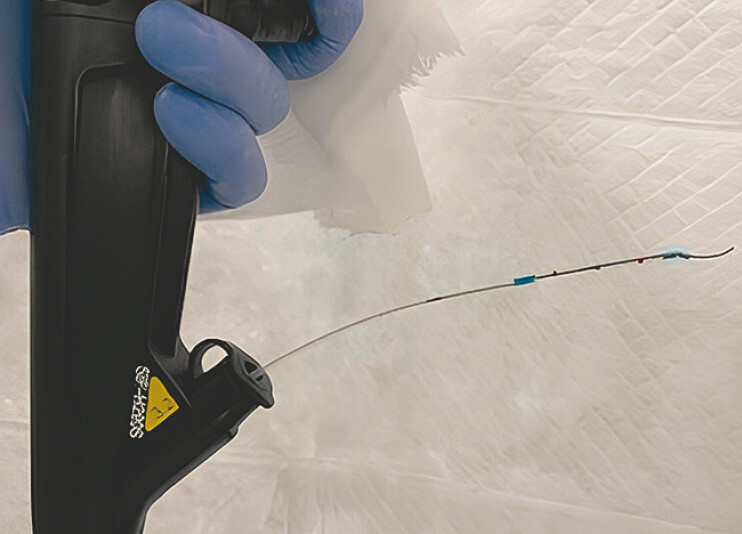
The catheter came out of the endoscopic channel.

**Fig. 5:**
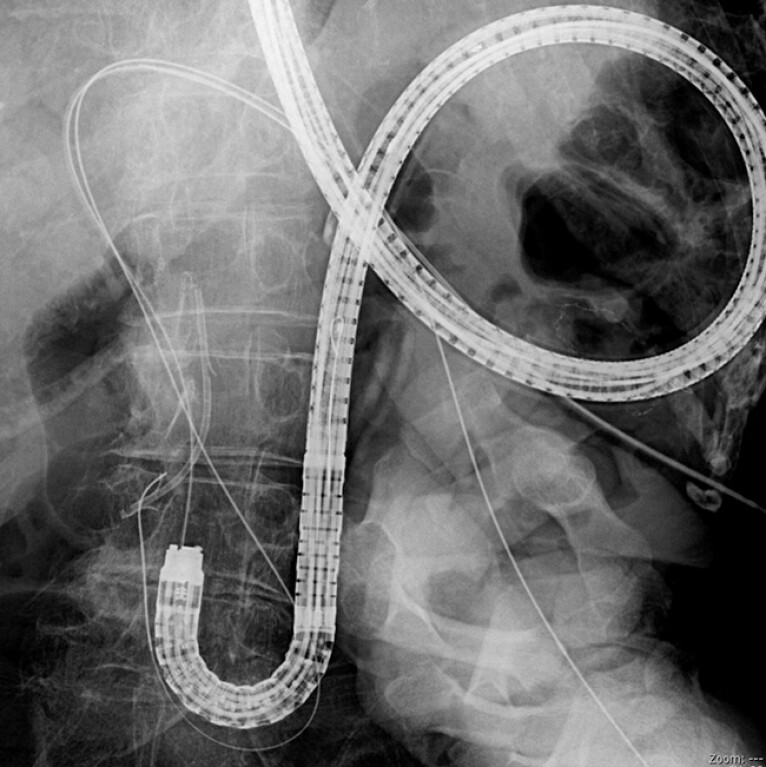
This image shows done rendezvous (CAS). CAS, catheter assistance system.

The two conventional rendezvous methods, (1) along-the-wire and (2) over-the-wire, entail some difficulties caused by possible miscannulation of the pancreatic duct, dropping of the grasped guidewire, and kinking in the endoscope channel 1 2 3 . By contrast, the CAS entails no such difficulties. Especially, when performing BE-ERCP, the CAS can support safer and more reliable rendezvous methods.

Endoscopy_UCTN_Code_CPL_1AH_2AJ

## Notes

Endoscopy E-Videos https://eref.thieme.de/e-videos
*E-Videos* is an open access online section of the journal *Endoscopy* , reporting on interesting cases and new techniques in gastroenterological endoscopy. All papers include a high-quality video and are published with a Creative Commons CC-BY license. Endoscopy E-Videos qualify for HINARI discounts and waivers and eligibility is automatically checked during the submission process. We grant 100% waivers to articles whose corresponding authors are based in Group A countries and 50% waivers to those who are based in Group B countries as classified by Research4Life (see: https://www.research4life.org/access/eligibility/ ). This section has its own submission website at https://mc.manuscriptcentral.com/e-videos .
